# Serological Detection of SARS-CoV-2 IgG Using Commercially Available Enzyme Immunoassays on Dried Blood Spots Collected from Patients

**DOI:** 10.1128/Spectrum.01245-21

**Published:** 2021-12-15

**Authors:** Gregory J. Walker, Rebecca Davis, Zin Naing, Brad McEntee, Yonghui Lu, Tatijana Denadija, William D. Rawlinson

**Affiliations:** a Virology Research Laboratory, Prince of Wales Hospital, Sydney, New South Wales, Australia; b School of Medical Sciences, Faculty of Medicine, University of New South Wales, Sydney, New South Wales, Australia; c Serology and Virology Division, NSW Health Pathology, Prince of Wales Hospital, Sydney, New South Wales, Australia; d Department of Microbiology and Infectious Diseases, NSW Health Pathology, Royal Prince Alfred Hospital, Camperdown, New South Wales, Australia; e Special Health Accommodation, Sydney Local Health District, Camperdown, New South Wales, Australia; f School of Biotechnology and Biomolecular Sciences, Faculty of Science, University of New South Wales, Sydney, New South Wales, Australia; City University of Hong Kong

**Keywords:** COVID-19, DBS, diagnostics, dried blood spot, SARS-CoV-2, serology, virology

## LETTER

Serological methods for severe acute respiratory syndrome coronavirus 2 (SARS-CoV-2) antibody detection are routinely performed on plasma or serum from peripherally collected venous blood. This can be inconvenient and often unacceptable to children. Dried blood spots (DBSs) collected by finger prick or heel prick are a minimally invasive alternative previously used to detect antibodies against Epstein-Barr virus, HIV, hepatitis virus, rubella virus, and other viruses (1). Evaluation of DBSs for the detection of SARS-CoV-2 antibodies with commercially available enzyme immunoassays (EIAs) was performed by analyzing paired DBS and serum samples from 54 subjects between 5 and 73 years of age (see the supplemental material).

Whole-blood samples were collected from patients via venipuncture and spotted onto DBS cards (Whatman 903 Protein Saver cards), with the remainder put into a serum tube. DBS samples were then processed (see the supplemental material) and tested in parallel with matched serum samples for antibodies against SARS-CoV-2 using the spike-based Architect SARS-CoV-2 IgG II quantitative assay (research use only; Abbott Laboratories, Abbott Park, IL, USA) and the S1 spike- and nucleocapsid-based anti-SARS-CoV-2 enzyme-linked immunosorbent assay (ELISA) (Euroimmun, Lübeck, Germany), following the manufacturers’ instructions. The Architect SARS-CoV-2 IgG II quantitative assay is a chemiluminescent microparticle assay (CMIA) and records data as arbitrary units (AU), which were interpreted as positive (≥50 AU/ml) or negative (<50 AU/ml). For Euroimmun ELISAs, the ratio of sample absorbance (read at 450nm, reference 620nm) to that of the calibrator (optical density [OD] ratio) was used to categorize sample results as positive (OD of ≥1.1), equivocal (OD of ≥0.8 to <1.1), or negative (OD of <0.8). Samples returning an equivocal result were excluded from sensitivity and specificity calculations in this analysis (Euroimmun spike, *n* = 2; Euroimmun nucleocapsid, *n* = 9). Sensitivity and specificity were calculated using the results of parallel testing of peripherally collected serum samples as the reference standard. Concordance between quantitative measurements was calculated with Pearson’s correlation coefficient.

**FIG 1:**
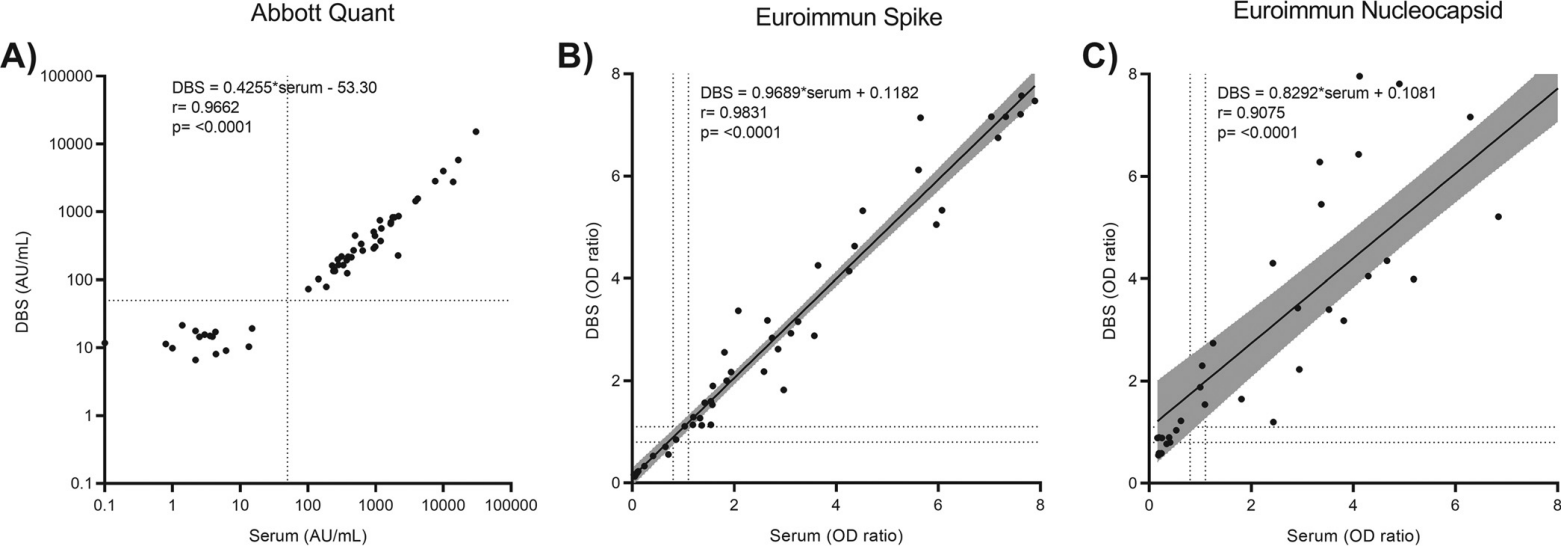
Linear regression of EIA OD ratios or AU per milliliter values for the detection of anti-SARS-CoV-2 IgG in 54 paired DBS and serum samples. Samples were tested with the Architect SARS-CoV-2 IgG II quantitative assay (A) and the Euroimmun anti-SARS-CoV-2 S1 spike ELISA (B) and nucleocapsid ELISA (subset, *n* = 46) (C). Dotted lines indicate the positive/negative, negative/equivocal, and equivocal/positive cutoff values for the respective assays. Shaded area on linear figures represents 95% confidence interval.

For the spike-based Architect and Euroimmun assays, there were no discordant results for any of the 54 paired samples. For both assays, DBS testing displayed 100% sensitivity and 100% specificity with reference to matched serum samples. The correlation coefficients between quantitative (Architect)/semiquantitative (Euroimmun) serum and DBS measurements were 0.9662 (95% confidence interval [CI], 0.9421 to 0.9803; *P* < 0.0001) and 0.9831 (95% CI, 0.9708 to 0.9902; *P* < 0.0001), respectively (Fig. 1). For a subset of 46 matched samples tested with the Euroimmun nucleocapsid-based assay, there was 1 discordant result, and DBS testing exhibited 100% sensitivity. One of 5 pairs that had a negative result in serum testing was positive in DBS testing. The correlation coefficient between quantitative serum and DBS measurements was 0.9075 (95% CI, 0.8380 to 0.9480; *P* < 0.0001).

The high sensitivity and specificity of DBS tests and the strong quantitative relationship with paired serum samples in the spike-based Architect and Euroimmun EIAs demonstrate the validity of DBS collection for the detection of SARS-CoV-2 antibodies with these assays. This is the first study to validate the quantitative Architect assay for DBS testing and the first to include pediatric specimens in the analysis. A limitation of this study was that whole blood from venipuncture was used for DBS collection instead of capillary blood, which would be used in practice. This was to prevent additional sampling from patients outside routine management. Our findings confirm those of recent studies in which adult DBSs (from capillary blood) were validated using semiquantitative EIAs targeting the S1 spike (2–4) and nucleocapsid proteins (3–5). These data suggest that DBS-derived blood is a viable alternative to plasma or serum collection. It has particular utility for testing of infants and children and testing in hotel or home quarantine, where sample collection is performed away from clinical settings. It can reduce transmission risks to staff members collecting samples from infected patients, as less time needs to be spent with the child to obtain the sample. Additionally, the simplicity of collection and processing and the stability of DBS samples for long periods at room temperature are well suited to coronavirus disease 2019 (COVID-19) diagnostics and surveillance in remote and resource-limited settings.

## References

[1] Amini F, Auma E, Hsia Y, Bilton S, Hall T, Ramkhelawon L, Heath PT, Le Doare K. 2021. Reliability of dried blood spot (DBS) cards in antibody measurement: a systematic review. PLoS One 16:e0248218. doi:10.1371/journal.pone.0248218. 33720928 PMC7959368

[2] Amendola A, Bianchi S, Gori M, Barcellini L, Colzani D, Canuti M, Giacomet V, Fabiano V, Folgori L, Zuccotti GV, Tanzi E. 2021. Dried blood spot as an alternative to plasma/serum for SARS-CoV-2 IgG detection, an opportunity to be sized to facilitate COVID-19 surveillance among schoolchildren. Pediatr Infect Dis J 40:e46–e47. doi:10.1097/INF.0000000000002955. 33181785

[3] Weisser H, Steinhagen K, Höcker R, Borchardt-Lohölter V, Anvari Ö, Kern PM. 2021. Evaluation of dried blood spots as alternative sampling material for serological detection of anti-SARS-CoV-2 antibodies using established ELISAs. Clin Chem Lab Med 59:979–985. doi:10.1515/cclm-2020-1436. 33554537

[4] Zava TT, Zava DT. 2021. Validation of dried blood spot sample modifications to two commercially available COVID-19 IgG antibody immunoassays. Bioanalysis 13:13–28. doi:10.4155/bio-2020-0289. 33319585 PMC7739400

[5] Mulchandani R, Brown B, Brooks T, Semper A, Machin N, Linley E, Borrow R, Wyllie D, EDSAB-HOME Study Investigators. 2021. Use of dried blood spot samples for SARS-CoV-2 antibody detection using the Roche Elecsys high throughput immunoassay. J Clin Virol 136:104739. doi:10.1016/j.jcv.2021.104739. 33588354 PMC7817498

