# Supplementary material for: Serological Detection of SARS-CoV-2 IgG Using Commercially Available Enzyme Immunoassays on Dried Blood Spots Collected from Patients
Source: Microbiol Spectr. 2021 Dec 15;9(3):e01245-21. doi: 10.1128/Spectrum.01245-21 (PMC8672893; doi:10.1128/Spectrum.01245-21)
Supplement: SUPPLEMENTAL FILE 1 — Supplemental material. Download SPECTRUM01245-21_Supp_1_seq5.pdf, PDF file, 0.3 MB [file spectrum01245-21_supp_1_seq5.pdf]

Serological detection of SARS-CoV-2 IgG using a commercially available enzyme immunoassays on dried blood spots collected from patients

Gregory J Walker, Rebecca Davis, Zin Naing, Brad McEntee, Yonghui Lu, Tatijana Denadija, William D Rawlinson

Corresponding author: Prof William Rawlinson, Virology Research Laboratory, Level 3 Clinical Sciences Building, Prince of Wales Hospital, Randwick 2031, Australia.

E-mail: w.rawlinson@unsw.edu.au Ph: +61 2 93829188

This file includes:

Supplementary methods. Participant recruitment

Supplementary methods. Processing of dried blood spot samples

Table S1. Age distribution of patients sampled

Participant recruitment

Patients under the care of the Sydney Local Health District (NSW, Australia) who required serology as part of routine management were identified for the study. This included 41 COVID-19 patients with a current RT-qPCR diagnosis and 13 who did not have a current positive diagnosis. Recruitment occurred during Feb 2021, prior to the vaccine rollout in Australia.

Processing of dried blood spot samples

Whole blood was collected from patients via venepuncture and spotted onto Whatman 903 Protein Saver DBS cards. DBS samples were then processed by methods applicable to each assay platform.

For the ARCHITECT SARS-CoV-2 IgG II Quantitative Assay (Abbott Laboratories, Abbott Park, IL, USA) a 6mm spot was punched using a standard office hole punch and transferred to a 2 ml round bottom centrifuge tube. After 300µl of 0.25% Tergitol 15-S-9 in PBS was added, the sample tube was incubated on an orbital shaker at 400 rpm for 60 min, followed by a centrifugation at 10,000g for 10 min. Supernatant (130µL) was then transferred to a sample cup and loaded onto ARCHITECT i2000SR analyzer for testing. As the current on-market ARCHITECT SARS-CoV-2 IgG II Quantitative Assay is not claimed for DBS use, a Research Use Only (RUO) assay file was installed to facilitate the DBS sample measurement. The DBS method for this platform may be further modified to improve agreement with quantitative serum results.

For the Euroimmun Anti-SARS-CoV-2 ELISA (Euroimmun, Lübeck, Germany) assays, a single 4.76mm disk was punched and incubated in 250µL of ELISA buffer (kit component) for one hour at 37°C. The eluted solution was then pipette mixed thoroughly and 100uL was taken forward for analysis as per the manufacturer's instructions.

Table S1. Age distribution of patients sampled

**Age**	**n=**
All	54
5-12	4
13-18	4
19-40	29
41-60	12
>60	5
